# Functional assessment of early retinal changes in diabetic patients without clinical retinopathy using multifocal electroretinogram

**DOI:** 10.1186/s12886-020-01677-6

**Published:** 2020-10-14

**Authors:** Mai A. Mohammed, Mohamed M. Lolah, Mohamed Fahmy Doheim, Amir AbouSamra

**Affiliations:** 1 Ophthalmology Department, Faculty of Medicine, Alexandria University, Alexandria, Egypt; 2 Faculty of Medicine, Alexandria University, Alexandria, Egypt

**Keywords:** Multifocal electroretinogram, Diabetic retinopathy, Diabetes duration, Amplitude, Implicit time

## Abstract

**Background:**

We aimed to assess early retinal changes in diabetic subjects without clinical retinopathy using multifocal electroretinogram (mfERG).

**Methods:**

Twenty eyes of 20 diabetic subjects type 2 without retinopathy and 20 eyes of 20 healthy controls of the same age and sex were eligible for our study and underwent mfERG. MfERG responses were recorded; N1–P1 amplitude and P1 implicit time of the 5 rings recorded were measured and analyzed.

**Results:**

The reduction in N1–P1 amplitude and the delay in P1-implicit time in type 2 diabetic subjects were statistically significant in most of the assessed rings compared to controls (*p* <  0.001). Moreover, N1–P1 amplitude was negatively correlated with diabetes duration. However, there was a positive correlation between P1-implicit time and diabetes duration in type 2 diabetic subjects in four out of five rings (p <  0.001).

**Conclusions:**

Our study showed reduced mfERG N1–P1 amplitude and delayed P1-implicit time indiabetic patients without retinopathy compared to normal controls. Implicit time andamplitude were significantly affected by diabetes duration. These results propose a valuable role of mfERG in evaluating the expected neuroretinal dysfunction before the clinical development of diabetic retinopathy. Early detection of functional abnormalities indicates that the patients need more tight medical control of diabetes. More well-designed studies are needed to assert upon these results.

## Background

Diabetes mellitus (DM) is a growing global disease that affects about 366 million patients worldwide with an increasing trend in developing countries [1, 2]. Diabetic retinopathy (DR) is a highly prevalent complication for DM patients. It affects almost one-third of DM patients and may be a leading cause of visual impairment among vital groups of population [3, 4]. There is an asymptomatic stage from developing DM to the appearance of clinical signs of DR in which clinically unnoticed microvascular changes and neural retinal damage occur and progress. Therefore, if the disease can be detected at an early stage, this could provide a better way of managing patients at a known risk of progressing DR and we may preserve the vision of a lot of people with DM for a longer time [5].

The previous evidence from published studies proposed that clinical vascular changes follow the neural changes occurring in the retina of those cases [6, 7]. Hence, several methods have been introduced to assess local retinal function in DM, and among them; studies have shown that multifocal electroretinogram (mfERG) may be a highly sensitive tool for detecting early neural dysfunction in the retina. Moreover, mfERG revealed that the implicit time increased significantly in the eyes of DM patients without DR compared to controls [8–11].

A study that generated a model using the abnormal mfERG for patients without DR and other risk factors demonstrated that mfERG can predict the beginning of DR in patients with DM [12]. Additionally, other studies also reported that, after a 3-year follow-up, the retinal areas with delays in the implicit time were at eightfold risk of DR development [13]. Hence, mfERG implicit time and amplitude may be considered as good predictors of DR in retinas with no retinopathy which can be a valid tool for clinicians and researchers with follow-up either through more tight diabetic control and trial of novel therapies that can delay or even prevent the progression of DR [8, 9].

Optical coherence tomography angiography (OCTA) defined retinal vascular and choriocapillaris parameters in diabetic patients without clinically evident DR. Hence, OCTA can detect microvascular changes that are not otherwise noted on clinical examination. These pre-clinical findings may facilitate earlier intervention for improved glycemic control and prevention of the onset of clinical retinopathy. However, neural dysfunction that is detected by mfERG precedes preclinical vascular changes that can be detected by OCTA which allows earlier intervention to protect the patients [14–16]. In this study, we aimed to assess the neural function of the retinal in diabetic patients using mfERG comparing its responses in DM patients without DR to healthy control participants from the Egyptian population.

## Method

This clinical study was approved by the local ethics committee of the Faculty of Medicine, Alexandria University, Egypt. The tenets of the declaration of Helsinki were followed and all patients signed informed consent.

According to ETDRS classification, we included subjects with diabetes for at least 2 years, but without DR. Our participants did not have any end-organ damage including renal damage and neuropathies with no other diabetes-related comorbidities. We obtained information about the type and by reviewing the medical records of the subjects. Fasting blood sugar (FBS) was measured within a few days of performing mfERG that give a clue about the recent status of patients’ diabetic control as glycosylated hemoglobin level could not be obtained in all patients and had a limitation in its performance and interpretations in our country. We excluded subjects with a history of any other ocular diseases that can affect mfERG results. Subjects with best-corrected visual acuity (BCVA) worse than 6/9 or visible media opacity or a high degree of refractive error (spherical equivalent> 6.00 DS of myopia and > 5.00 DS of hyperopia) were also excluded. Additionally, fundus fluroscein angiography and optical coherence tomography were done to exclude any features of diabetic retinopathy and the average central macular thickness was 280 μm with no evidence of macular atrophy or edema. Matched controls without diabetes of the same age and sex were selected that had no significant previous ocular history and was attending for a routine ocular check-up with normal fundus and average macular thickness was about 280 μm. Similar criteria were applied to them.

All participants underwent complete ophthalmic assessment including visual acuity and anterior segment examination by slit-lamp biomicroscopy, Bailey-Lovie Log MAR chart, refraction, intraocular pressure measurement, fundus examination together with fundus fluroscein angiography and optical coherence tomography before performing mfERG. There were no microaneurysms and the different assessments were free.

### Multifocal electroretinogram

We used the Roland system for recording mfERG according to the International Society for Clinical Electrophysiology of Vision (ISCEV) guidelines. The patients had a proper near correction during the mfERG examination. The stimuli were 61 scaled hexagons generated on a high-resolution color monitor. The viewing distance was set at 33 cm, which corresponded to a stimulated field of ±24° vertically and ± 30° horizontally. In order to be outside the frequency of recorded signals and to provide higher temporal resolution, a high frame frequency of 120 Hz was chosen. The neutral and reference electrodes were a large size and disposable placed on the frontocentral and external canthus, respectively. The cornea was anesthetized with topical 4% xylocaine. H k loop electrodes were used as an active electrode during recording mfERG. We measured N1–P1 amplitude from the first negative wave trough (N1) to the first positive wave peak (P1) while the time of P1 peak was considered P1-implicit time [17]. We analyzed the responses depending on regional averages from five concentric rings centered on the fovea (0°-2°, 2°-5°, 5°-10°, 10°-15°, and > 15°) in both study groups.

### Statistical analysis

Data were analyzed using SPSS 25.0 software. Number and percent were used for qualitative data while quantitative data were described using mean and standard deviation. Results were considered statistically significant at a *P*-value of less than 0.05. Considering that our data were not normally distributed, the *Mann*-Whitney test (non-parametric) was used to investigate the differences between the DM and control subjects regarding the mfERG responses. Using spearman’s correlation, we assessed the possible correlation of diabetes duration with mfERG amplitude and implicit time.

## Results

We considered mfERG data of 20 eyes of 20 DM subjects without DR and 20 eyes of 20 control participants for our analysis. All diabetic subjects were of type 2 diabetes. DM subjects’ mean age was 59.5 ± 5.3 years while the mean age of controls was 60.1 ± 5.4 years. Among 20 type 2 diabetic subjects, ten were males and the rest ten were females, and similarly were the controls. The mean duration of diabetes was 8.4 ± 5.4 years and the mean FBS level was 170.9 ± 25.8 mg/dL in diabetic subjects.

Our results showed that N1–P1 amplitude in all the rings of the retina was significantlydecreased between DM subjects compared to controls (*p* <  0.001) (Table 1).

**Table 1:** Mean N1–P1 amplitudes (nV/deg2) for 5 rings of retina

Area of retina	Type 2 diabetic subjects (*N* = 20)	Controls (N = 20)	*p* value
Ring 1	67.5 ± 28.4	103.4 ± 19.9	< 0.001
Ring 2	31.3 ± 14.6	87.8 ± 11.19	< 0.001
Ring 3	16.1 ± 4.8	78.5 ± 12.2	< 0.001
Ring 4	7.8 ± 3	57.2 ± 7.3	< 0.001
Ring 5	5.2 ± 2.6	51.2 ± 6.4	< 0.001

Similarly, we found that P1-implicit time in all the rings of the retina was significantly increased between DM subjects compared to controls (Table 2) except for ring 2 (*P* = 0.40).

**Table 2:** Mean P1-implicit times (ms) for 5 rings of retina

Area of retina	Type 2 diabetic subjects	Controls	*p* value
Ring 1	50.5 ± 4.3	47.6 ± 1.4	0.009
Ring 2	48.6 ± 2.6	47.6 ± 4	0.405
Ring 3	46.8 ± 3.1	43.7 ± 1.5	< 0.001
Ring 4	47.1 ± 3.2	43.1 ± 1.1	< 0.001
Ring 5	47.3 ± 4.2	42.7 ± 1.2	< 0.001

The results showed an association of mfERG amplitude with diabetes duration and FBS level in the type 2 DM group with a similar association for the implicit time. N1–P1 amplitude in retinal rings showed a significant negative correlation with diabetes duration in all the rings except ring 4 (Table 3). Moreover, P1- implicit time in retinal rings showed a significant positive correlation with diabetes duration in all the rings except ring 2 (Table 4).

**Table 3:** Correlation of N1–P1 amplitudes with diabetes duration in type 2 diabetic subjects

Areas of retina	Correlation (r)	*p* value
Ring 1	−0.731	< 0.001
Ring 2	−0.761	< 0.001
Ring 3	−0.804	< 0.001
Ring 4	−0.285	0.223
Ring 5	−0.605	0.005

**Table 4:** Correlation of P1-implicit time with diabetes duration in type 2 diabetic subjects

Areas of retina	Correlation (r)	*p* value
Ring 1	0.632	0.003
Ring 2	0.347	0.134
Ring 3	0.621	0.003
Ring 4	0.559	0.01
Ring 5	0.681	0.001

## Discussion

The results of our study revealed that mfERG N1–P1 amplitude was reduced and P1- implicit time was increased in type 2 DM subjects without DR compared to normal controls that were consistent with the previously published studies. Although studies reported showed that implicit time is affected more in DM patients and is a better indicator of developing clinical DR than the amplitude [9, 10, 18], our results were consistent with Adhikari et al. [5] which reported that both of them may be affected in type 2 DM patients. In those aforementioned studies, the minimal decreased amplitude, but highly increased implicit time can be explained by showing the bipolar cells function. It is known to be the primary initiators of N1–P1 amplitudes and a part of the inner retina with the photoreceptors and could be impaired in DM subjects without DR, but it is not entirely stopped [19, 20]. However, significantly decreased amplitude in our population may be explained by clear loss of function for the bipolar cells and photoreceptors before the beginning of any clinical DR in our population which could be explained by marked uncontrolled blood sugar level.

The reported results on the relation of diabetes duration and BFS level with ERG response are controversial. For instance, a study that did not use multifocal but rather used global flash stimulus instead, demonstrated longer durations of diabetes may be associated with a marginal propensity for ERG amplitudes [11]. However, Adhikari et al. [5] reported that both average N1–P1 amplitude and average P1-implicit time did not show any significant correlation with the FBS level. Moreover, another study by Kim et al. [21] showed that the DM duration and glucose control condition does not affect mfERG response locally. In this study, N1–P1 amplitude was negatively correlated with diabetes duration while there was a positive correlation between P1-implicit time and diabetes duration in type 2 DM subjects in four out of five rings.

Our study had encountered some limitations. We did not use HB A1c and instead ofthat FBS was used. Despite HbA1c is known to be more accurate than FBS for assessingdiabetic subjects over a longer period of time, we measured FBS within a few days of performing mfERG that give a clue about the recent status of patients’ diabetic control as HbA1c could not be obtained in all patients and we had a limitation regarding its performance and interpretations in our country. Moreover, some data related to the duration of diabetes was obtained from the medical records of patients so it might not have been fully reliable and this might have affected those results.

## Conclusion

To recapitulate, our study showed reduced mfERG N1–P1 amplitude and delayed P1-implicit time in diabetic patients without retinopathy compared to normal controls. Implicit time and amplitude were significantly affected by diabetes duration. These results propose a valuable role of mfERG in evaluating the expected neuroretinal dysfunction before the clinical development of diabetic retinopathy. Early detection of functional abnormalities indicates that the patients need more tight medical control of diabetes. More well-designed studies are needed to assert upon these results.

## Data Availability

The data used to support the findings of this study are available from the corresponding author upon request.

## Abbreviations

DM: Diabetes mellitus
mfERG: Multifocal electroretinogram
DR: Diabetic retinopathy
FBS: Fasting blood sugar
BCVA: Best-corrected visual acuity
ISCEV: Clinical Electrophysiology of Vision
